# Efficacy of Transcranial Direct Current Stimulation on Pain Level and Disability of Patients with Fibromyalgia: A Systematic Review of Randomized Controlled Trials with Parallel-Group Design

**DOI:** 10.3390/brainsci14010026

**Published:** 2023-12-25

**Authors:** Anita Azarkolah, Ahmad Ali Noorbala, Sahar Ansari, Amir-Homayun Hallajian, Mohammad Ali Salehinejad

**Affiliations:** 1 Imam Khomeini Hospital, Tehran University of Medical Sciences, Tehran P.O. Box 1416634793, Iran; 2 Psychosomatic Medicine Research Center, Tehran University of Medical Sciences, Tehran P.O. Box 1416634793, Iran; 3 Department of Psychology, University of Tehran, Tehran P.O. Box 14155-6456, Iran; 4 Department of Psychology and Neurosciences, Leibniz-Institut für Arbeitsforschung, 44139 Dortmund, Germany; 5 School of Cognitive Sciences, Institute for Research in Fundamental Sciences (IPM), Tehran P.O. Box 1956836613, Iran

**Keywords:** transcranial direct current stimulation, noninvasive brain stimulation, fibromyalgia, pain, systematic review, RCT

## Abstract

Transcranial direct current stimulation (tDCS) has been increasingly applied in fibromyalgia (FM) to reduce pain and fatigue. While results are promising, observed effects are variable, and there are questions about optimal stimulation parameters such as target region (e.g., motor vs. prefrontal cortices). This systematic review aimed to provide the latest update on published randomized controlled trials with a parallel-group design to examine the specific effects of active tDCS in reducing pain and disability in FM patients. Using the PRISMA approach, a literature search identified 14 randomized controlled trials investigating the effects of tDCS on pain and fatigue in patients with FM. Assessment of biases shows an overall low-to-moderate risk of bias. tDCS was found effective in all included studies conducted in patients with FM, except one study, in which the improving effects of tDCS were due to placebo. We recommended tDCS over the motor and prefrontal cortices as “effective” and “probably effective” respectively, and also safe for reducing pain perception and fatigue in patients with FM, according to evidence-based guidelines. Stimulation polarity was anodal in all studies, and one single-session study also examined cathodal polarity. The stimulation intensity ranged from 1-mA (7.14% of studies) to 1.5-mA (7.14% of studies) and 2-mA (85.7% of studies). In all of the included studies, a significant improvement in at least one outcome variable (pain or fatigue reduction) was observed. Moreover, 92.8% (13 of 14) applied multi-session tDCS protocols in FM treatment and reported significant improvement in their outcome variables. While tDCS is therapeutically effective for FM, titration studies that systematically evaluate different stimulation intensities, durations, and electrode placement are needed.

## 1. Introduction

Fibromyalgia (FM) is a chronic rheumatic condition characterized by widespread musculoskeletal pain, physical exhaustion, and cognitive difficulties [1]. The primary hallmark of FM is pain, often accompanied by symptoms like fatigue, anxiety, depression, and catastrophic thinking, which collectively diminish the overall quality of life for affected individuals [1]. The global prevalence is estimated at approximately 2% to 4% in the general population, and the prevalence exceeds 15% in selected clinical samples [2]. This condition predominantly affects women. Currently, there is no definitive objective method established for detecting and diagnosing FM, and diagnosis relies on criteria outlined by the American College of Rheumatology [3].

The pathophysiology of FM is not fully understood; however, there is clear evidence of the involvement of pain pathways in FM, and there is a strong association with central sensitization, known as an excessive response to painful stimulation secondary to altered nociception at the central nervous system [4]. Additionally, there is evidence of central nervous system alterations in FM, which are mostly linked to a deficiency in inhibitory control. This is demonstrated through atypical cortical excitability, characterized by reduced short intracortical inhibition and facilitation in the motor cortex [5]. There is also evidence of an association between FM and altered resting state functional connectivity in the dorsolateral prefrontal cortex (DLPFC), which is mainly responsible for cognitive control, including inhibitory control [6]. Furthermore, studies show that the DLFPC is a critical component of the neural circuit involved in processing the cognitive and emotional aspects of pain [7].

Recent technological advances in cognitive and clinical neuroscience focus on specific non-pharmacological treatments that can regulate cortical excitability, thereby influencing the functions of the central nervous system in order to modulate the descending pain inhibitory system, potentially impacting pain relief. Noninvasive brain stimulation (NIBS) methods, like transcranial direct current stimulation (tDCS), are safe, affordable, and potent neuromodulatory approaches for therapeutic purposes in neuropsychiatric and neurological disorders [8,9,10]. Previous studies have shown that tDCS is effective in alleviating pain in various chronic pain conditions, including FM [11,12,13]. What makes the application of tDCS and other noninvasive brain stimulation techniques promising in FM is the underlying pathophysiology, which is related to brain functional and structural abnormalities. In addition to brain functional abnormalities, FM comes with related cognitive and affective deficits [14,15,16]. Modulating cortical (and subcortical) activities with tDCS is assumed to regulate such functional abnormalities and, hopefully, associated cognition and behavior.

Although the application of tDCS in FM has grown in recent years, the number of standard tDCS studies with robust experimental designs is still limited and warrants further investigation. Furthermore, there are questions about the efficacy of tDCS in FM treatment and the optimal target region for stimulation. At present, there is limited but suggestive evidence in support of the pain-relieving impact of tDCS when compared to a sham treatment of FM, although there is notable variability in the results [11]. Accordingly, there is a need to update the current literature on the efficacy of tDCS for FM. The most recent systematic review of this topic included studies published until June 2022 and included studies without control and placebo groups as well [17]. One novel aspect of the current systematic review compared to recent reviews and meta-analyses [11,17,18] is that we only included randomized controlled trials that applied tDCS (no other electrical stimulation and no combined intervention) in a parallel-group design with a sham control group. This was to examine the sole and specific effects of active tDCS in reducing pain in FM patients and to prevent potential carry-over effects that might occur in studies with a cross-over design. We used the Preferred Reporting Items for Systematic Reviews and Meta-Analyses (PRISMA) approach to systematically review the latest randomized-controlled trials that applied tDCS to date in patients with FM. The purpose of this systematic review was (1) to evaluate the efficacy of tDCS in reducing primary and secondary pain in patients with FM, (2) to examine efficacy of tDCS in improving disability (i.e., fatigue) in patients with FM, and (3) to investigate the superiority of stimulation over the motor vs. prefrontal cortices in reducing pain in patients with FM. We hypothesized that tDCS would have a significant analgesic effect on FM pain when compared with sham tDCS.

## 2. Methods

### 2.1. Information Resources, Search Strategy, and Eligibility Criteria

**Figure 1:**
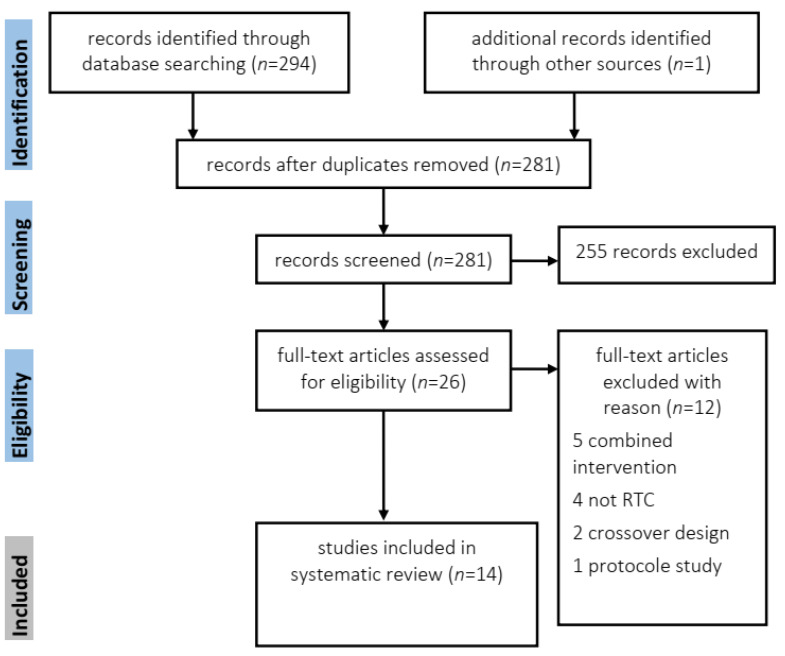
PRISMA flow diagram of included studies investigating the effects of transcranial direct current stimulation on reducing pain in patients with fibromyalgia.

Using the PRISMA guidelines [19], we conducted a systematic search, performed by the first author, in datasets including PubMed (Medline), Scopus, and Web of Science. We used the following search terms: (‘Fibromyalgia’ OR ‘Widespread chronic pain’ OR ‘Pain’ OR ‘pain disorder’ OR ‘chronic fatigue syndrome’) AND (‘transcranial electrical stimulation’ OR ‘transcranial direct current stimulation’ OR ‘tES’ OR ‘tDCS’) with the final search updated in August 2023. We applied no year limit to the search. We additionally checked review articles, meta-analyses, and relevant book chapters for cross-references. The PRISMA flow diagram is shown in Figure 1.

### 2.2. Study Inclusion

We included only peer-reviewed published randomized controlled trials in our analysis. The inclusion criteria were: (1) studies with randomized controlled trial design and a sham (placebo) group condition; (2) studies published in international peer-reviewed journals and in English; (3) studies that were conducted on patients with fibromyalgia (studies conducted on healthy subjects were excluded) and which measured clinical pain. The exclusion criteria included: (1) studies with a crossover design; (2) studies with incomplete outcome data reporting; (3) protocol papers; (4) studies employing a qualitative methodology; (5) studies that used a combined intervention with transcranial direct current stimulation (tDCS) and other treatments. The final search identified a total of 294 studies. After removing duplicates and screening the abstracts based on the inclusion criteria, 26 RCTs remained for full-text assessment and data extraction. 12 studies were excluded with reasons (Figure 1) for using combined intervention with tDCS (*n* = 5) [20,21,22,23,24], not being an RCT *(n* = 4), crossover design (*n* = 2) [25,26], and one protocol study (*n* = 1) [27]. 14 studies were thus included in the final analysis [28,29,30,31,32,33,34,35,36,37,38,39,40,41].

### 2.3. Outcome Variables

Measures of clinical pain and measures of disability were the primary and secondary outcome variables. Clinical pain was measured with the Numeric Rating Scale (NRS), including the Visual Numeric Scale [42,43], Visual Analog Scale (VAS) [44], 36-item short-form survey (SF-36) for pain [45], and the Pain Catastrophizing Scale (PCS) [46]. The disability measure was the Fibromyalgia Impact Questionnaire (FIQ) [47], which is a brief 10-item, self-administered instrument that measures physical functioning, work status, depression, anxiety, sleep, pain, stiffness, fatigue, and well-being, and the Short Form-36—physical functioning (SF-36-PF) [48].

### 2.4. Risk of Bias

We performed the risk of bias assessment using the Cochrane Collaboration’s tool [49]. Specifically, for each study, authors evaluated the risk of selection (random assignment, allocation concealment), performance (blinding of participants and examiners), detection (blinding of outcome measures), attrition (incomplete outcome data), reporting, and other biases. The risk of bias can be categorized as low, high, or uncertain, as shown in Table 1.

## 3. Results

### 3.1. Risk of Bias

The risk of bias for each tDCS study on FM is reported in Table 1. Of fourteen studies, only one study used a single-blind design [35], yielding a potential detection bias as the experimenter was not blind to the tDCS condition. One recent study also benefited from a triple-blind design [40]. The percentage of studies with selection bias, reporting bias, performance bias, detection bias, and attrition bias are summarized in Table 1 at 3 levels: low, uncertain, and high risk of bias. Overall, there is around 57% of uncertain selection bias in the included studies which was mostly due to unclear randomization and allocation concealment. Three studies also rated with high risk of bias with respect to incomplete/missing outcome data [34,35,37]. Overall, the assessment of the risk bias of the studies was satisfactory.

### 3.2. Overview of tDCS RCTs in FM

Details of the 14 tDCS RCTs in FM, including study design, stimulation parameters, sample size, outcome measures, and major findings, are summarized in Table 2. In what follows, we give a brief overview of the targeted outcome measures and important parameters of tDCS interventions applied to FM patients, especially the target region, stimulation intensity, and repetition rate.

#### 3.2.1. tDCS over the Primary Motor Cortex

A total of 6 of 14 studies (42.8%) solely targeted the primary motor cortex in patients with FM [30,32,33,34,37,40]. In all of these studies, the active tDCS group significantly reduced pain scores on different measures (e.g., VAS, FIQ, FAS, SF36-pain) as well as fatigue scores. In those studies that had a follow-up, pain reduction was observed up to 90 days after the intervention [40]. In the studies that also measured mood and quality of life, depression scores and quality of life were improved after the intervention [34,40]. The intensity of tDCS in all of these studies was 2 mA except in one study with 1 mA [33], and stimulation polarity in all the studies was anodal. The return electrode placement was on the right supraorbital area in four studies, or on the contralateral arm in one study. In all the studies, tDCS was applied in a multi-session design from 5 to 10 consecutive days. The duration of stimulation per session in all studies was 20 min except for one study which applied a novel duration (13 min stimulation—20 min break—13 min stimulation) [40]. The rationale for applying this duration was that multiple-spaced stimulation periods have been shown to facilitate tDCS-based interventions, and long periods of stimulation (20+ min) might lead to an undesirable involvement of hemostatic brain mechanisms that can limit the increase in plasticity [40]. Details of these studies, including stimulation protocols, sample size, outcome measures, and major findings, are summarized in Table 2 and supplementary Figure S1. Overall, the results of these studies suggest improving effects of tDCS on pain level and fatigue scores of patients with FM.

#### 3.2.2. tDCS over the DLPFC

A total of 3 of 14 studies solely applied tDCS over the DLPFC [35,36,38]. In all of these studies, an anodal electrode was placed over the left DLPFC (F3), and the return electrode (cathodal) was placed over the right DLPFC (F4), based on the standrad 10–20 EEG system. The stimulation intensity was 1.5 mA in one study and 2 mA in two studies. In all studies, stimulation was repeated over either 8, 20, or 60 days, and stimulation duration was 20 min in two studies and 30 min in the other. Details of these studies, including stimulation protocols, sample size, outcome measures, and major findings, are summarized in Table 2 and supplementary Figure S1. All of the studies show that repeated tDCS over the left DLPFC significantly reduced pain level/scores (measured by VAS, FIQ, PCS) as compared to the sham group. In one of the studies [35], tDCS was applied to the occipital region in another group of patients. The results of this study showed that repeated sessions of DLPFC tDCS significantly improved pain as well as fatigue, while stimulation of the occipital region only improved pain level.

#### 3.2.3. tDCS over M1 vs. DLPFC

Of the 14 included studies, four RCTs examined the effects of tDCS over the primary motor cortex and DLPFC in different groups of patients [28,29,39,41], which allows us to compare the efficacy of each protocol. In all of the studies, stimulation intensity was 2 mA, and stimulation polarity was anodal. Stimulation duration was for 20 min, which was delivered over 5, 10, 15, or 30 days. The return electrode was Fp2 in the M1 protocol and F4 or Fp2 in the DLPFC protocols, based on the standrad 10–20 EEG system. Details of these studies, including stimulation protocols, sample size, outcome measures, and major findings, are summarized in Table 2 and supplementray Figure S1.

In one of the studies [28], it was shown that tDCS over the motor cortex resulted in significantly greater pain improvement compared with sham stimulation and stimulation of the DLPFC. In another study, although both M1 tDCS and DLPFC tDCS resulted in improvements in pain scores and quality of life at the end of the treatment protocol, only stimulation of the motor cortex resulted in long-lasting benefits at 30 and 60 day follow-ups [29]. In a recently published study [39], the effects of active tDCS over the M1, DLPFC, and insular cortex were compared with sham stimulation, and pain and fatigue score follow-up was measured up to 6-months. This study found significant treatment effects across time for clinical pain and for fatigue, cognitive and sleep disturbances, and experimental pain, irrespective of the group, which provides evidence of a placebo effect. The only outcome measure that was specific to tDCS was mood, which was significantly improved in both M1 tDCS and DLPFC tDCS groups [41]. The most recently published RCT also compared the efficacy of M1 vs. DLPFC tDCS on the pain and fatigue states of patients with FM after 20 sessions of stimulation. This study found that anodal tDCS over the DLPFC significantly reduced pain scores by 36.53% compared to 25.79% in sham tDCS, while a-tDCS on the M1 reduced pain scores by 45.89 compared to the sham group. A similar response pattern was observed on the disability scale in the groups that received anodal tDCS compared to sham tDCS over the M1 and DLPFC, with larger effects on the M1 protocol. Also, this study found a higher reduction in serum brain-derived neurotrophic factor (BDNF) from baseline to treatment end that was positively correlated with decreased pain scores regardless of the treatment group.

#### 3.2.4. Other Cortical Regions

Only in three studies were regions other than the primary motor cortex and DLPFC stimulated, and these regions were the supra-orbital region [31], occipital region [35], and the operculo-insular cortex [39]. The first study found significant pain reduction in cathodal and anodal supra-orbital region groups, although this study examined the effect of single-session tDCS (anodal/cathodal stimulation of supra-orbital vs. motor cortex). One important aspect of this study was the modeling of the electrical current in tDCS montages. The authors found that electrode montage is a critical factor to consider in the clinical application of tDCS for pain control as there is an important correlation between the location of induced electrical current and tDCS-induced analgesic effects [31]. In the second study [35], eight repeated sessions of occipital tDCS with 1.5 mA intensity improved pain, but not fatigue. Finally, the last study found the treatment effect of tDCS over the operculo-insular cortex across time for clinical pain and fatigue which was the same for the sham group and other active tDCS groups, suggesting a placebo effect [39].

#### 3.2.5. Home-Based tDCS

One important aspect of the included studies was the use of home-based tDCS for clinical use in patients with pain, which was investigated in three studies during and after the COVID-19 pandemic [36,38,41]. While in two studies only DLPFC tDCS was applied [36,38], in one study both M1 tDCS and DLPFC tDCS were compared. All of the studies found significant improving effects of home-based tDCS in the pain level of patients with FM, providing evidence that HB-a-tDCS is a viable and effective therapeutic approach. Details of these studies, including study design, stimulation parameters, sample size, outcome measures, and major findings, are summarized in Table 2 and previous sections of the results.

## 4. Discussion

In this systematic review, we investigated the efficacy and the randomized-controlled trials that applied tDCS to patients with FM. A novel aspect of this systematic review was to include studies that applied tDCS (no other electrical stimulation and no combined intervention) in a parallel-group design. This was to examine the specific effects of active tDCS in reducing pain in FM and to prevent potential carry-over effects that might occur in studies with a cross-over design. With regard to efficacy, and regardless of size of effect and target region, tDCS was effective in 100% of the RCTs with a parallel-group design conducted on patients with FM. Only in one study were the improving effects of active tDCS due to placebo, as similar effects were shown in the sham group [39]. Stimulation polarity was anodal in all studies, and one single-session study also examined cathodal polarity. The stimulation intensity ranged from 1 mA (7.14% of studies) to 1.5 mA (7.14% of studies) and 2 mA (85.7% of studies). In all of the variables included, a significant improvement effect on at least one of the outcome variables (pain or fatigue reduction) was observed. Moreover, 92.8% (13 of 14) applied multi-session tDCS protocols for FM and reported significant improvement in their outcome variables. Assessment of the biases of the included studies shows that there is a need for the prevention of selection bias, especially with respect to allocation concealment, but this risk was not high. In what follows, we discuss several methodological considerations that are important for the clinical efficacy and feasibility of tDCS in FM.

### 4.1. Target Region

The two brain regions that were the most targeted in the majority of RCTs of FM were the primary motor cortex and the DLPFC. Although some studies suggested larger effects of M1 tDCS vs. DLPFC tDCS, results of a new meta-analysis show that comparing studies with M1 and DLPFC stimulation sites did not show differences in the effect of tDCS on pain [11], supporting the analgesic effect of both protocols. There are different explanations for the pain-reducing effects of stimulating both M1 and DLPFC. Modulating activity of the M1 would result in the modulation of motor cortex excitability, which influences aspects of sensory pain processing and ultimately enhances the descending pain inhibitory system [13]. On the other hand, targeting the DLPFC would lead to an adjustment of the cognitive and emotional aspects of pain due to its connections with limbic system structures [6,50]. One point to consider here is the focality of tDCS in its traditional form (large electrodes) and the approximate location of the M1 and DLPFC. This suggests that targeting the M1 or DLPFC could lead, therefore, to the simultaneous modulation of several pain processing and affective/cognitive pathways [11]. Another relevant explanation for efficacy of DLPFC tDCS for FM is the comorbidity of depressive states in FM patients [51] and the effectiveness of DLPFC tDCS for improving mood [52,53,54,55]. It is possible that the effects of DLPFC tDCS in reducing pain in FM patients could be partially due to its mood-improving effects. In addition to the M1 and DLPFC, the occipital region and operculo-insular cortex were also targeted in two studies, and their relevance to pain modulation was not more than the DLPFC or M1. One important methodological aspect is to apply tDCS over the motor cortex at the patient’s preferred time of day and under no sleep pressure, as these factors are shown to significantly affect motor cortical excitability and tDCS-induced plasticity in the motor cortex [56,57].

### 4.2. Efficacy

In accordance with the latest evidence-based guidelines, we can evaluate the efficacy of the applied tDCS protocols in reducing pain levels in FM patients. Accordingly, anodal M1 tDCS can be categorized as “effective” in reducing pain in patients with FM [8]. The findings from recent robust RCT studies, including those by Caumo et al. (2023) [41] and Samartin-Veiga et al. (2022) [39], also indicate a “probably effective” use of DLPFC tDCS in pain reduction in patients with FM. This emerging evidence underscores the necessity of revisiting and potentially updating the guidelines for tDCS application to FM to incorporate these new insights for more effective clinical application.

### 4.3. Combined Intervention

One rationale behind this systematic review was to examine the efficacy of tDCS alone in reducing pain and fatigue in FM patients. This is why we excluded five RCTs that used other interventions combined with tDCS. This, however, should not ignore the therapeutic effects of combined protocols, which might indeed be larger than tDCS alone, and we briefly discuss it here. Applying tDCS combined with aerobic exercise resulted in a greater reduction of levels of pain, anxiety, and mood in patients with FM and was shown to be superior to each intervention alone [22]. Another study that combined tDCS with functional exercise, however, found that pain intensity, psychological symptoms, and quality of life increased significantly in both groups that received exercise alone and exercise with tDCS [24]. In another study, DLPFC tDCS was applied concurrently with working memory training in FM patients. Here, the authors found that combining both techniques resulted in specific cognitive effects on short-term and long-term episodic memory and executive functions, which has clinical relevance for top-down treatment approaches in FM [23]. In another recent study, a combination of tDCS with low-dose Naltrexone was explored. The combined protocol was not superior but had benefits in reducing pain frequency and intensity [21]. Finally, the efficacy of tDCS combined with occipital nerve stimulation was examined in patients with FM, and it was found that adding bifrontal tDCS to occipital nerve stimulation has no added benefit in improving fibromyalgia-related symptoms [20]. These studies do not provide strong evidence for the larger efficacy of combining tDCS with another intervention; however, this needs to be systematically investigated in larger trials.

### 4.4. Limitations of the Studies

The two main types of limitations associated with tDCS studies in FM include those related to protocol and those related to study design. The most common design-related limitation is the number of subjects in the active group, which was limited (sample size ≥ 20) in 6 of 14 studies. Only three of the included studies have a sample size larger than 30 in the group that received active tDCS. This is especially important for evaluating the clinical efficacy of the intervention. With regard to protocol parameters, the included studies were mostly consistent with respect to target region (M1 and DLPFC), stimulation polarity (anodal), and duration (20 min per session). There is, however, still a need to systematically investigate different stimulation parameters (e.g., different intensities, duration, electrode placement, etc.) in one homogeneous sample size. With regard to the target region, future study designs comparing M1 and DLPFC and their effects on different dimensions of pain are needed to address the effects of each region on pain perception. This is particularly interesting for applying multi-channel protocols with smaller electrodes in which both promising target regions can be stimulated simultaneously.

### 4.5. Conclusions

Taken together, current research provides strong evidence for the therapeutic application of tDCS over both the primary motor and cortex and DLPFC for reducing pain and fatigue in patients with FM. Furthermore, the relative ease of access and the portability of tDCS devices for both clinical and home-based treatments [58] suggest potential utility in addressing pain-related issues in various clinical scenarios, such as with cancer patients. Given the increased prevalence of FM in cancer patients [59,60] and the co-occurrence of depressive symptoms and pain, targeting the DLPFC with tDCS could be beneficial [61,62]. This approach may offer a dual therapeutic effect by potentially improving mood and reducing pain and fatigue. Such possibilities, while promising, warrant cautious exploration in future research to better understand their implications in the management of comorbid conditions in oncological and broader clinical contexts. That said, we still need large-scale RCTs and translational studies that can investigate a wide range, from basic neurophysiology to applications in cognitive-clinical neuroscience, in order to establish the clinical efficacy of tDCS in FM. Inter-individual variabilities should also be considered, in line with a “personalized” approach in non-invasive brain stimulation research.

## Figures and Tables

**Table 1:** Bias assessment for included tDCS studies on fibromyalgia (*n* = 14) [28,29,30,31,32,33,34,35,36,37,38,39,40,41] using the Cochrane risk of bias tool.

	Fregni et al. (2006) [28]	Valle et al. (2009) [29]	Riberto et al. (2011) [30]	Mendonca et al. (2011) [31]	Fagerlundet al. (2015) [32]	Jales et al. (2015) [33]	Khedr et al. (2017) [34]	To et al. (2017) [35]	Brietzke et al. (2020) [36]	De Melo et al. (2020) [37]	Caumo et al. (2022) [38]	Samartin-Veiga et al. (2022) [39]	Loreti et al. (2023) [40]	Caumo et al. (2023) [41]	+ Low Risk of Bias
															? Uncertain Risk of Bias
															− High Risk of Bias
*Selection bias*: **Random sequence generation**	**?**	**?**	**?**	**?**	**+**	**?**	**+**	**?**	**+**	**+**	**+**	**+**	**+**	**+**	57.14% low, 42.8% uncertain, 0% high
*Selection bias:* **Allocation concealment**	**+**	**?**	**?**	**+**	**?**	**+**	**?**	**?**	**+**	**?**	**+**	**+**	**+**	**+**	57.14% low, 42.8% uncertain, 0 % high
*Reporting bias*: **Selective reporting**	**+**	**−**	**+**	**+**	**+**		**+**	**+**	**+**	**+**	**+**	**+**	**+**	**+**	92.9% low, 0% uncertain, 7.1% high
*Performance bias*: **Blinding (participants and personnel)**	**+**	**+**	**+**	**+**	**+**	**+**	**+**	**?**	**+**	**+**	**+**	**+**	**+**	**+**	92.9% low, 7.1% uncertain, 0% high
*Detection bias*: **Blinding of outcome assessment**	**+**	**+**	**+**	**+**	**+**	**+**	**+**	**?**	**+**	**?**	**+**	**+**	**+**	**+**	85.8% low, 14.2% uncertain, 0% high
*Attrition bias*: **Incomplete outcome data**	**+**	**+**	**+**	**+**	**+**	**+**	**−**	**−**	**+**	**−**	**+**	**+**	**+**	**+**	78.6% low, 0% uncertain, 21.4% high
*Other bias*	**+**	**+**	**+**	**+**	**+**	**+**	**−**	**+**	**+**	**−**	**+**	**+**	**+**	**+**	85.8% low, 0% uncertain, 14.2% high

**Table 2:** Summary of tDCS studies in patients with fibromyalgia.

#	Author	Design (Control Condition)	*n* Active/Sham	Mean Age ± SD	Target Electrode Site	Return Electrode Site	Electrode Size	Intensity	Session Number and Duration	Polarity	Pain Outcome Measure	Major Finding
1	Fregni et al. (2006) [28]	RCT double blind (sham controlled)	Active = 22 Sham = 11	53.4 ± 8.9	Left dlPFC/ M1	Fp2	7 × 5 cm	2 mA	5 × 20 min	anodal	VAS/SF36-PF	Anodal tDCS of the primary motor cortex induced significantly greater pain improvement compared with sham stimulation and stimulation of the DLPFC.
2	Valle et al. (2009) [29]	RCT double blind (sham controlled)	Active = 27 Sham = 14	54.8 ± 9.6	Left dlPFC/ M1	Fp2	7 × 5 cm	2 mA	10 × 20 min	anodal	VAS/FIQ	M1 and DLPFC stimulation both display improvements in pain scores and quality of life at the end of the treatment protocol. Only M1 stimulation resulted in long-lasting benefits at 30 and 60 follow-ups.
3	Riberto et al. (2011) [30]	RCT double blind (sham controlled)	Active = 11 Sham = 12	58.3 ± 12.1	M1	Fp2	7 × 5 cm	2 mA	10 × 20 min	anodal	SF36 (pain)/FIQ	Active treatment had a significantly greater reduction of SF-36 pain domain scores and a tendency toward higher improvement in FIQ scores as compared with sham tDCS.
4	Mendonca et al. (2011) [31]	RCT double blind (sham controlled)	Active = 24 Sham = 6	43.2 ± 9.8	supra-orbital region/ M1	transition of the cervical and thoracic spine	8 × 10 cm	2 mA	1 × 20 min (4 single sessions)	anodal/cathodal	VNS/PPT	significant pain reduction in cathodal and anodal supra-orbital region groups indexed by VNS.
5	Fagerlund et al. (2015) [32]	RCT double blind (sham controlled)	Active = 24 Sham = 24	N/A	M1	Fp2	7 × 5 cm	2 mA	5 × 20 min	anodal	NRS/FIQ	A small, significant improvement in pain was observed under the active tDCS but not the sham condition. Fibromyalgia-related daily functioning improved in the active tDCS group vs. the sham group.
6	Jales et al. (2015) [33]	RCT double blind (sham controlled)	Active = 10 Sham = 10	46.4 ± 10.61	M1	Fp2	7 × 5 cm	1 mA	10 × 20 min	anodal	VAS/SF36-PF	decrease in the Fibromyalgia Impact Questionnaire and the Visual Analog Scale scores in the active tDCS
7	Khedr et al. (2017) [34]	RCT double blind (sham controlled)	Active = 18 Sham = 18	31.3 ± 10.9	M1	Contralateral arm	6 × 4 cm	2 mA	10 × 20 min	Anodal	VAS	Higher improvement in the experimental scores of the patients in the real tDCS group in VAS, pain threshold and depressive scores
8	To et al. (2017) [35]	RCT single blind (sham controlled)	Active = 15 Sham = 16	46.95 ± 10.07	Left dlPFC/ occipital region	O2—F4	NR	1.5 mA	8 × 20 min	Anodal	NRS/PCS/MFIS	Repeated sessions of occipital tDCS improved pain, but not fatigue, whereas repeated sessions of DLPFC tDCS significantly improved pain as well as fatigue
9	Brietzke et al. (2020) [36]	RCT double blind (sham controlled) HB	Active = 10 Sham = 10	48.6	Left dlPFC	F4	7 × 5 cm	2 mA	60 × 30 min	Anodal	VAS/FIQ	After the first 20 sessions of a-tDCS, the cumulative pain scores reduced by 45.65% vs. 3.94 and at the end of 60 sessions by 62.06% vs. 24.92% in active vs. sham tDCS, respectively.
10	De Melo et al. (2020) [37]	RCT double blind (sham controlled)	Active = 13 Sham = 13	44.81 ± 8.8	M1	NR	7 × 5 cm	2 mA	5/10 × 20 min	Anodal	VAS/CIRS	Reduction in pain intensity after treatment for groups in general in addition to a reduction in alpha 2 oscillations in the frontal and parietal after 5 days
11	Caumo et al. (2022) [38]	RCT double blind (sham controlled) HB	Active = 32 Sham = 16	49.06 ± 9	Left dlPFC	F4	7 × 5 cm	2 mA	20 × 20 min	Anodal	VAS/FIQ/PCS	a-tDCS reduced the Pain Catastrophizing Scale total scores by 51.38% compared to 26.96% in s-tDCS, and the Profile of Chronic Pain: Screen total scores by 31.43% compared to 19.15% in s-tDCS
12	Samartin-Veiga et al. (2022) [39]	RCT double blind (sham controlled)	Active = 100 Sham = 30	50.31 ± 8.76	M1/dlPFC/operculo-insular cortex	Fp2	NR	2 mA	15 × 20 min	Anodal	VAS/FIQ	Significant treatment effects across time for clinical pain and for fatigue, cognitive and sleep disturbances, and experimental pain
13	Loreti et al. (2023) [40]	RCT triple blind (sham controlled)	Active = 17 Sham = 18	41.99 ± 10.16	M1	Fp2	7 × 5 cm	2 mA	10 × 13 min 20 min with 13 min break	Anodal	VAS/FAS	The active tDCS group showed improvement in pain after 10, 30, and 90 days compared with the sham tDCS. Improvement in quality of life (QoL) and fatigue was observed in the active tDCS group
14	Caumo et al. (2023) [41]	RCT double blind (sham controlled) HB	Active = 68 Sham = 34	46.96 ± 9.42	M1/left dlPFC	F4—Fp2	7 × 5 cm	2 mA	20 × 20 min	Anodal	FIQ/PCS	a-tDCS on DLPFC significantly reduced pain scores by 36.53% compared to 25.79% in s-tDCS. a-tDCS on M1 reduced pain scores by 45.89% compared to 22.92% over s-tDCS.

Note: **tDCS** = transcranial direct current stimulation; **RCT** = randomized controlled trial; SD = standard deviation; **dlPFC** = dorsolateral prefrontal cortex; **M1** = primary motor cortex; **F4** = right dorsolateral prefrontal cortex; **Fp2** = right supraorbital area; **VAS** = Visual Analogue Scale; **FIQ** = Fibromyalgia Impact Questionnaire; **VNS** = Visual Numeric Scale; **PPT** = Pain Pressure Threshold; **PCS** = Pain Catastrophizing Scale; **MFIS** = Modified Fatigue Impact Scale; **NRS** = Numeric Rating Scale; **SF36** = Short-Form 36 Health Questionnaire; **CIRS** = Cumulative Illness Rating Scale; **FAS** = Fatigue Assessment Scale; **HB** = home-based; **NR** = not reported or available.

## Data Availability

Data sharing is not applicable to this systematic review article. The data that support the findings are available in the manuscript.

## Supplementary Materials

The following supporting information can be downloaded at: https://www.mdpi.com/article/10.3390/brainsci14010026/s1, Table S1: checklist; Figure S1: Summary of stimulation parameters of included studies. Note: in a and c the number of applied protocols in 14 studies, some of which included both M1 and DLPFC tDCS, are calculated. tDCS = transcranial direct current stimulation; DlPFC = dorsolateral prefrontal cortex; M1 = pri-mary motor cortex.
